# Meta-Analysis of Cognitive Performance in Neurodevelopmental Disorders during Adulthood: Comparisons between Autism Spectrum Disorder and Schizophrenia on the Wechsler Adult Intelligence Scales

**DOI:** 10.3389/fpsyt.2020.00187

**Published:** 2020-03-26

**Authors:** Susan S. Kuo, Shaun M. Eack

**Affiliations:** ^1^Department of Psychology, University of Pittsburgh, Pittsburgh, PA, United States; ^2^School of Social Work and Department of Psychiatry, University of Pittsburgh, Pittsburgh, PA, United States

**Keywords:** intelligence, general cognition, nonsocial cognition, transdiagnostic, cross-diagnosis, development, Asperger syndrome, psychosis

## Abstract

Autism Spectrum Disorder (ASD) and schizophrenia are neurodevelopmental disorders which show substantial cognitive heterogeneity in adulthood, yet it remains unclear whether cognitive profiles may overlap across these diagnoses. Thus, the aim of this review was to summarize comparisons between ASD and schizophrenia on nonsocial cognition in adulthood. To minimize between-study heterogeneity in a relatively small literature, subtest scaled scores from the Wechsler Adult Intelligence Scale were compared between ASD (*N*=190) and schizophrenia (*N*=260) in six studies comprising a total of 450 participants. Meta-analyses of 11 subtests indicated that participants with ASD demonstrated significantly better performance than schizophrenia for visuospatial perception and reasoning and problem solving (Hedge’s *g*=0.636), as well as visual attention and organization (*g*=0.433-0.475). Participants with ASD also demonstrated better performance than those with schizophrenia for working memory (*g*=0.334) and language (*g*=0.275), and generally comparable performance on processing speed and verbal comprehension. These findings were largely stable across age, sex, intelligence quotient (IQ), intellectual disability, scale version, and age- and sex-matching. Overall, ASD and schizophrenia showed striking differences in visuospatial perception and reasoning and problem solving, small differences in working memory and language, and substantial overlap in processing speed and verbal comprehension. These cognitive profiles were generally stable from adolescence to middle adulthood. To our knowledge, this is the first review to summarize comparisons of nonsocial cognition in verbal adults with ASD or schizophrenia. These findings are consistent with and substantially extend prior meta-analyses of case-control studies for ASD and schizophrenia (8, 9), which also suggest that, in comparison to neurotypical controls, ASD demonstrates smaller cognitive impairments than schizophrenia across most cognitive domains, particularly working memory, visuospatial learning/memory, and language. Our findings therefore highlight the importance of comparing cognition transdiagnostically to inform the etiologies of these neurodevelopmental disorders and to refine shared and unique targets for remediating cognition.

## Introduction

Autism spectrum disorder (ASD) and schizophrenia are both neurodevelopmental disorders which show substantial difficulties with both social and nonsocial information processing (1, 2). Although these two disorders have different peak ages of onset, with ASD emerging early in childhood (3) and schizophrenia emerging in late adolescence and early adulthood (4), their neurodevelopmental trajectories may be associated with similar cognitive impairments during adulthood (5–7). Recent meta-analyses of cognitive functioning in adulthood within these two disorders indicate that both conditions show deficits compared to typically developing adults in the same cognitive domains, including processing speed, attention and vigilance, working memory, visuospatial learning/memory, verbal learning/memory, language, and reasoning and problem-solving (8, 9). However, no review to date has investigated direct comparisons of nonsocial cognition in adulthood between these two disorders. Given that nonsocial cognition is an important predictor of functional outcomes in both disorders (10, 11), comparing nonsocial cognition between ASD and schizophrenia is critical for adapting common strategies for remediating cognition to improve functional outcomes across these neurodevelopmental disorders.

Although the domains of cognitive impairments appear to be overlapping in ASD and schizophrenia, little evidence to date bears directly upon whether the magnitudes of these impairments are comparable across disorders. Thus far, most studies comparing cognitive functioning across ASD and schizophrenia have investigated social cognitive abilities such as theory of mind and emotion processing (12). Meta-analyses of these studies suggest that ASD and schizophrenia demonstrate relatively similar performance across social cognitive domains (12). Although 19 studies were included in this meta-analysis, findings for each social cognitive domain were informed by three to eight studies which each contributed largely non-overlapping measures. Thus, meta-analytic comparisons are tempered by substantial method heterogeneity across the contributing studies.

Relatively fewer studies have examined the extent to which nonsocial cognitive abilities may overlap or differ from each other across ASD and schizophrenia (7, 13, 14). These studies have largely drawn upon standardized cognitive batteries, the most common of which are the Wechsler Adult Intelligence Scales [WAIS; (15, 16)]. To our knowledge, only one study has compared nonsocial cognition between ASD and schizophrenia using a cognitive battery that does not include the WAIS (7). Thus, although there is a smaller pool of studies that compare nonsocial cognitive performance across ASD and schizophrenia compared to the number of studies that compare social cognitive performance across these diagnoses, the common use of the WAIS to assess nonsocial cognitive performance reduces method heterogeneity compared to the use of different batteries across studies.

Beyond studies that directly compare nonsocial cognition between ASD and schizophrenia, the most relevant support for shared and distinct features in nonsocial cognition across ASD and schizophrenia come from recent comprehensive meta-analyses comparing cognitive performance within these disorders to neurotypical controls. Across domains, verbal adults with ASD demonstrate comparable performance to neurotypical controls for attention and vigilance and working memory but show cognitive impairments particularly for social cognition (Hedge’s *g*=-0.80 to -1.09), followed by nonsocial cognitive processes including processing speed (*g*=-0.61), verbal learning and memory (*g*=-0.55), and reasoning and problem-solving (*g*=-0.51) (9). In comparison, adults with first-episode schizophrenia, who have had less exposure to psychotropic medication relative to adults who are later in their schizophrenia course, show substantial difficulties across all social and nonsocial cognitive domains relative to neurotypical controls, with impairments ranging from 0.6 to 1.4 standard deviations below that of neurotypical performance (8). Mirroring cognitive deficits that have been implicated in ASD, cognitive domains that are impacted in schizophrenia include processing speed, perception, attention/vigilance, working memory, episodic memory, verbal learning, visual learning, executive functioning, affective processing, and social cognition (17, 18).

Overall, verbal adults with ASD appear to show deficits in specific cognitive domains and comparable performance to controls in other cognitive domains, consistent with a multiple-deficit model (19, 20). In contrast, adults with schizophrenia demonstrate widespread cognitive deficits across most cognitive domains, consistent with a generalized cognitive deficit (21). Across cognitive domains meta-analyzed within each diagnosis (8, 9), deficits appear more pronounced in schizophrenia than in ASD, with differences in overall effect size estimates approximating 0.3 to 0.6 standard deviations between the two conditions. Notably, the effect size estimates overlap for cognitive domains including processing speed, attention/vigilance, reasoning and problem solving, and social cognition, but do not overlap for working memory, visuospatial learning/memory, and language. Taken together, relative to neurotypical controls, ASD demonstrates smaller cognitive impairments than schizophrenia across most cognitive domains, with discrepancies being most evident for working memory, visuospatial learning/memory, and language.

The primary aim of this meta-analysis was to examine the extent to which ASD and schizophrenia show overlapping and unique impairments in nonsocial cognition on the most widely used cognitive battery for assessing domain functioning across these disorders, the WAIS (15, 16). We aimed to compare specific cognitive functioning across studies in a relatively small literature while simultaneously minimizing the heterogeneity in sensitivity and specificity across different cognitive measures that may inform a given cognitive domain. We therefore decided to adopt a conservative approach to consolidate effect size estimates from studies of nonsocial cognition across ASD and schizophrenia. Specifically, consolidating estimates for a given subtest from similar versions of a standardized battery reduces heterogeneity across studies and increases the precision of meta-analytical estimates relative to comparisons combining different cognitive measures. Supporting the utility of this approach, the theoretical, psychometric, and administrative consistencies across WAIS versions facilitate valid comparisons of differences in cognitive domain performance across ASD and schizophrenia. To our knowledge, this is the first study to systematically review nonsocial cognitive domain performance across ASD and schizophrenia.

## Methods

### Search Strategies

Peer-reviewed journal articles were screened to meet all of the following inclusion criteria:

1. The full-text article was published in English.
2. Participants were diagnosed based on criteria listed in the Diagnostic and Statistical Manual of Mental Disorders (version III-R or IV) (22, 23) or International Classification of Diseases (version 9 or 10) (24, 25).
3. The ASD group included only verbal individuals with a primary diagnosis of autism, high-functioning autism, or Asperger syndrome.
4. The schizophrenia group included only individuals with a primary diagnosis of schizophrenia, schizophreniform disorder, or schizoaffective disorder.
5. Subtest performance was reported by group for multiple subtests from a standardized Wechsler Adult Intelligence Scale.

**Figure 1:**
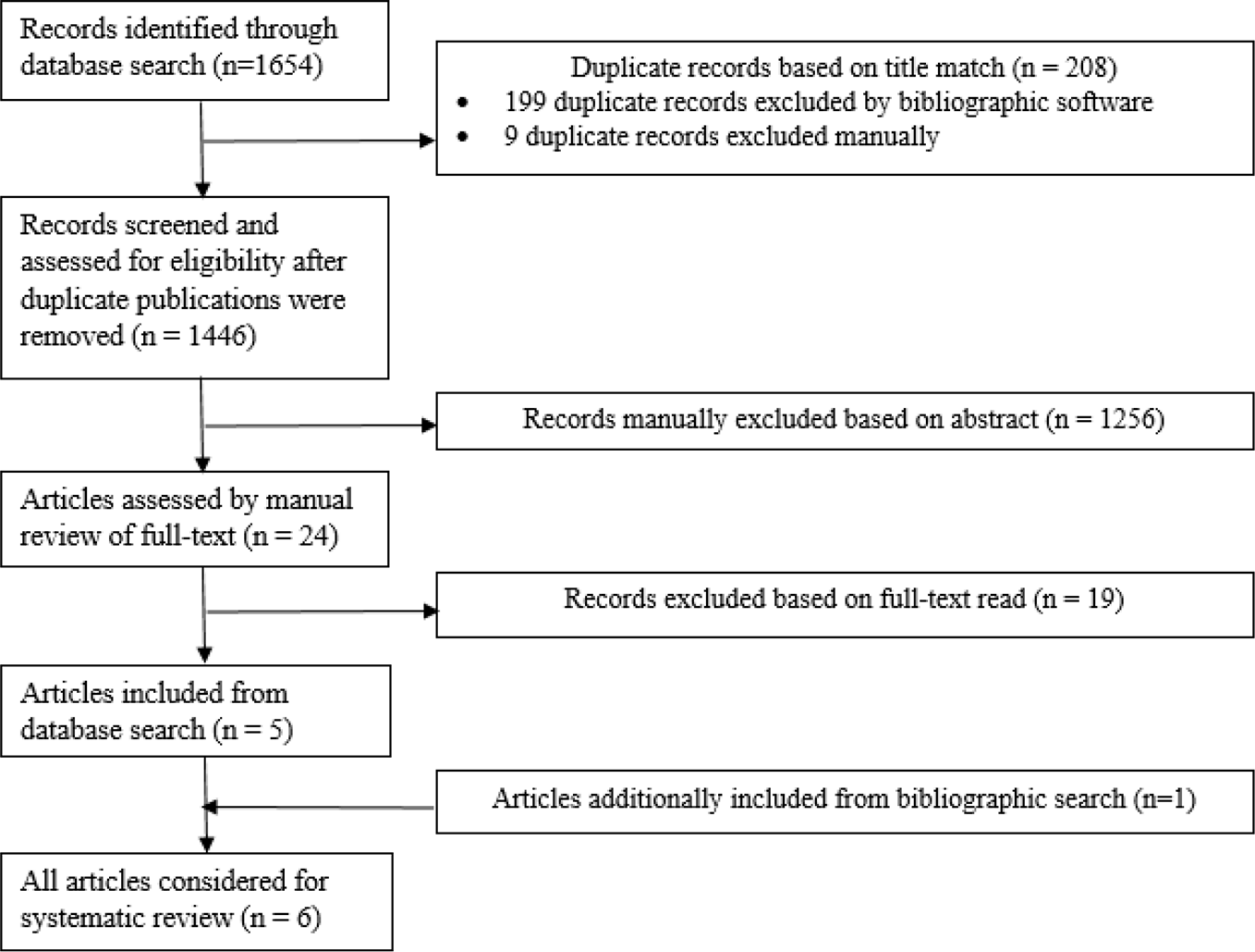
Flowchart depicting systematic review process.

As depicted in **Figure 1**, 1654 peer-reviewed journal articles published before February 15, 2019 were electronically identified using a conjunction of the following free-text search terms, “schizophreni*”, “autis* OR Asperger”, and “Wechsler” in two comprehensive journal databases, PubMed (*n*=33 articles) and PsycINFO (*n*=1621 articles). Duplicate publications were removed using EndNote X8 bibliographic software yielding 1446 articles. A total of 24 journal articles were examined after abstract, and 19 articles were not included after full-text read because they did not include subtest scores. Six articles met all inclusion criteria and were included in this systematic review after full-text read.

### Selection of Cognitive Domains

The WAIS versions identified in this meta-analysis have age-based norms from large, population-based normative samples with ages ranging from approximately 16 to 90 years (15, 16). We examined all WAIS subtests with at least four contributing studies. Thus, a total of 11 subtests were meta-analyzed. The WAIS is comprised of four different indexes, representing four cognitive domains (15, 16).

For Verbal Comprehension, which encompasses language as well as verbal reasoning, four subtests were included:

1. Similarities: this subtest prompts the participant to describe how two words or concepts are similar, to assess verbal reasoning;
2. Information: this subtest prompts the participant to describe their understanding of widely-known factoids, to assess general knowledge;
3. Vocabulary: this subtest prompts the participant to define the meanings of terms, to assess verbal knowledge, verbal expression, and concept formation;
4. Comprehension: this supplemental subtest prompts the participant to describe how they integrate and adapt to social information, to assess verbal reasoning and social inference.

For Perceptual Reasoning, which encompasses visuospatial abilities and reasoning and problem solving, two subtests were included:

1. Block Design: this timed subtest prompts the participant to recreate spatial patterns using blocks, to assess spatial reasoning;
2. Picture Completion: this timed supplemental subtest prompts the participant to identify missing parts in pictures, to assess attention, visual perception and organization.

For Working Memory, which encompasses attention and working memory, two subtests were included:

1. Digit Span: this subtest prompts the participant to verbally repeat strings of numbers forwards or backwards, to assess auditory working memory, attention, and concentration;
2. Arithmetic: this subtest prompts the participant to solve arithmetic problems presented as stories, to assess auditory working memory, concentration, and quantitative reasoning.

For Processing Speed, one subtest was included:

1. Digit Symbol Coding: this subtest prompts the participant to assess psychomotor speed, motor coordination, visual perception, attention, and concentration.

Finally, two additional subtests were included:

1. Object Assembly: this supplemental subtest prompts the participant to solve visual puzzles, to assess visual anticipation, visual perception, and motor reasoning;
2. Picture Arrangement: this timed supplemental subtest prompts the participant to order scrambled series of cards depicting social events, to assess nonverbal reasoning, sequencing and social inference.

Interestingly, in a confirmatory analysis study of all 14 WAIS-III subtests, the last two subtests, Picture Arrangement and Object Assembly, and Picture Completion, comprise a Social Cognition domain (26), suggesting that these subtests may be associated with social cognitive abilities.

### Data Extraction

The scaled score mean and standard deviation for each subtest for each group was extracted from the included studies and entered on two occasions by the first author. Scaled scores are age-adjusted and standardized to a sample distribution with a mean of 10 and a standard deviation of 3 (range=1-19), with higher scores indicating better performance. Where data were only available for subgroups of a schizophrenia sample in one study (27), we pooled the subgroup estimates to derive an overall estimate for the schizophrenia sample.

### Meta-Analysis

Random-effect meta-analyses of cognitive subtest performance were performed using the R package, metafor (28), weighting the studies by their inverse variance, which reflects the study sample size. We used Hedge’s *g* as the bias-corrected effect size of mean group differences. To characterize subtest performance within groups, we used the R package, meta (29), to estimate the random-effect inverse-variance weighted mean and standard error of performance within groups. Random-effect models were estimated instead of fixed-effect models due to the requirement that the true effect size does not vary between studies and the substantial Type I bias in significance tests for mean effect sizes and moderators in fixed-effects models (30).

### Heterogeneity

To quantify the heterogeneity in effect sizes across studies, we computed the *I^2^* (31), which is the percentage of variation across studies that is due to heterogeneity rather than chance. We further assessed potential publication bias attributable to small studies with Egger’s regression test (ERT) (32), which investigates correlations between effect sizes and sample sizes. A significant ERT may indicate that the effect size estimate may be biased by a selection of small sample studies. For subtests with significant mean group differences, we also examined whether the effect size estimates may be biased by the file-drawer problem by calculating a fail-safe number, which is the number of studies with null results that would have to be added to the current set of studies to raise the significance level of the effect size to *p=*.05 (33). We also conducted sensitivity analyses eliminating the only contributing study that included adolescents in addition to adults (13). This study assessed participants as young as 14 years using the Wechsler Intelligence Scale for Children-Revised [WISC-R; (34)] and reported results combining the WISC-R scores with the WAIS-R scores (13).

### Meta-Regression

For significant findings, we examined whether the effect sizes were moderated by key sample characteristics, including age (mean sample age), sex (mean sample proportion of males), intellectual disability (inclusion or exclusion of participants who had an estimated IQ<70; in addition, one study in Turkey included participants who had ≥12 years of education as a proxy for lack of intellectual disability, as eligibility for post-secondary education is based on standardized test performance assessing cognitive abilities (35), and scale version (WAIS, WAIS-III, or WAIS-R). Although the scaled scores for each participant were based on age-based norms, we also examined whether the effect sizes were moderated by mean group differences in age and sex. Education was reported in only three studies (14, 27, 35), and was therefore not examined as a possible moderator. As clinical and functioning characteristics of the groups were not reported consistently across studies, we were unable to examine the effects of these potential moderators. Given the number of meta-regression analyses conducted, we adopted a conservative cutoff for statistical significance at *p*<0.01.

## Results

### Sample Characteristics

Descriptive characteristics of the included studies are presented in **Table 1**. The number of included WAIS measures for each study ranged from 4 to 14 (median=11), and a minimum of 375 participants contributed to each subtest meta-analysis. All studies were cross-sectional and included at least 13 participants in each diagnostic group, with mean group sizes of 43 participants in the ASD groups and 32 participants in the schizophrenia groups.

**Table 1:** Description of studies included in meta-analysis reporting cognitive domain performance in autism and schizophrenia, sorted by increasing mean sample age.

Characteristic	Group	Mean Across Studies	Bölte, Rudolf (13)	Goldstein, Minshew (27) ^ǂ^	Marinopoulou, Lugnegård (14)	Murphy (36)	Mançe Çalişir, Atbaşoğlu (35)	de Boer, Spek (37)
Test Version			WISC-R, WAIS-R	WAIS-R	WAIS-III	WAIS-R	WAIS ^	WAIS-III
Number of Included Measures		10	11	11	14	4	7	14
*N*	ASD	43	20	31	50	13	32	114
	SZ	32	20	80	33	13	17	27
Recruitment	ASD		N/A	N/A	Outpatient clinic and adult rehabilitation records	Forensic psychiatric hospital	N/A	Mental health institution
	SZ		University hospital inpatient and outpatient clinics	Veterans’ hospital inpatient clinic	Outpatient clinic	Forensic psychiatric hospital	Newspaper advertisement	Mental health institution
Diagnosis	ASD		Autism	High-functioning autism excluding Asperger syndrome	Asperger syndrome	Asperger syndrome	Autism and Asperger syndrome	High-functioning autism and Asperger syndrome
	SZ		Schizophrenia	Schizophrenia	Schizophrenia, schizoaffective disorder, schizophreniform disorder	Schizophrenia	Schizophrenia	Schizophrenia
Age (years)	ASD	28.2 (7.8)	16.8 (2.1)	21.4 (9.8)	27.7 (3.9)	32.1 (6.5)	33.9 (9.4)	37.4 (10.6)
	SZ	28.4 (9.1)	16.6 (1.5)	–	29.1 (4.3)	30.2 (4.2)	24.6 (3.2)	41.5 (9.3)
Sex (% male)	ASD	68%	55%	–	50%	100%	53%	81%
	SZ	73%	55%	100%	55%	100%	47%	78%
Education	ASD		–	10.7 (2.9)	14% some college	–	16.1 (2.6)	–
	SZ		–	–	21% some college	–	13.4 (1.1)	–
Intellectual Disability			No exclusion ^+^	Excluded IQ<70	Excluded IQ<70	No exclusion	Excluded education <12 years	Excluded IQ<80
Estimated IQ	ASD	98.0 (8.9)	82.5 (24.1)	99.6 (13.1)	102.4 (12.3)	100.1 (15.9)	–	105.3 (12.5)
	SZ	90.8 (9.1)	83.9 (22.3)	–	94.5 (13.4)	82.9 (8.3)	–	101.9 (12.3)

ASD, autism spectrum disorder; SZ, schizophrenia. N/A, not available. WAIS-III: Wechsler Adult Intelligence Scale-Third Edition (15); WAIS-R: Wechsler Adult Intelligence Scale-Revised (16); WISC-R: Wechsler Intelligence Scale for Children-Revised (34).

^ǂ^ Goldstein, Minshew (27) divided schizophrenia into four clusters: Moderately Impaired, High Functioning, Severely Impaired, and Severely Impaired Psychomotor.

^+^30% of ASD group has comorbid epilepsy.

^Scale version is Turkish translation of the first scale version (38) due to difficulties with norming for subsequent editions.

#### Age

Across studies, groups were matched for mean age (*t*(4)=0.519, *p=*.631). Participant groups had mean ages ranging from 16 to 41, allowing for comparisons of age-related differences in cognitive functioning from adolescence to middle adulthood, with the group mean ages approximating 28.2 years of age (*S.D.*=7.8 years).

#### Sex

Across studies, groups were not evenly matched for sex ratios (*χ^2^*(10)=82.535, *p*<0.001), with a smaller proportion of males in the ASD groups compared to the SZ groups. In line with epidemiological estimates of sex ratios for ASD and schizophrenia during early through middle adulthood (4, 39), all the participant groups, except for a schizophrenia group in one study, included more males than females (mean group proportion ~70% male).

#### IQ

Across studies, IQ was within overlapping range between groups (*t*(3)=1.711, *p=*.186). All studies except for two (13, 36) excluded participants who had an IQ lower than 70 or had fewer than 12 years of education. Overall, all except one study reported an estimated full-scale IQ, with mean group IQs being approximately 98.0 for the ASD groups and 90.8 for the schizophrenia groups.

### Diagnostic Comparisons of Language and Verbal Comprehension

**Figure 2:**
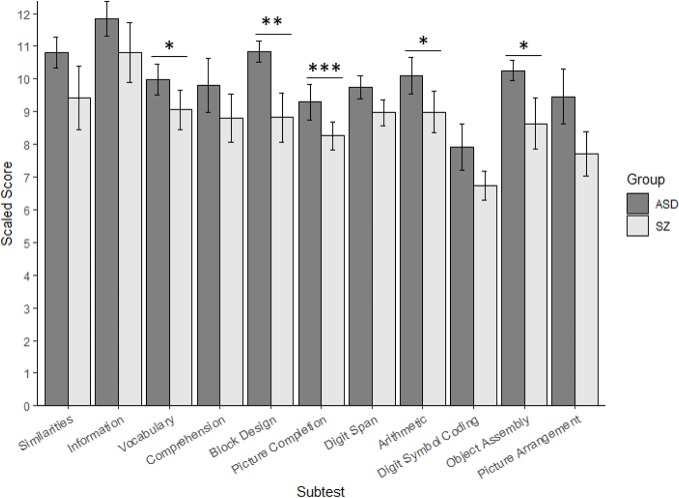
Cognitive Performance on the Wechsler Adult Intelligence Scale in Autism and Schizophrenia. ASD, autism spectrum disorder; SZ, schizophrenia. Random-effects, inverse-variance weighted subtest means and standard errors calculated for each group are presented with the significance of the meta-analytic effect size (the bias-corrected group mean difference). Although standard errors may overlap between groups for a given subtest, the effect size may be significant given that the effect sizes are bias-corrected, and vice versa. *p<.05; **p<.01; ***p<.001.

**Table 2** presents the results of the meta-analyses for each WAIS subtest, whereas **Figure 2** depicts the random-effects, inverse-variance weighted subtest means and standard errors calculated within each group.

**Table 2:** Summary of meta-analyses comparing mean group differences in cognitive functioning between autism and schizophrenia.

Cognitive Domain	Number of Studies	Combined ASD *n*	Combined SZ *n*	ASD Scaled Score	SZ Scaled Score	Effect Size	Effect Size 95% C.I.	Effect Size *p*-value	*I^2^*	ERT (*p*-value)	Fail-Safe *n*
VCI: Similarities	6	260	190	10.81 (0.47)	9.42 (0.97)	0.389	(-0.061, 0.839)	.090	77.0	1.027 (.305)	–
VCI: Information	5	247	177	11.84 (0.54)	10.81 (0.90)	0.287	(-0.034, 0.608)	.079	53.5	-0.797 (.425)	–
VCI: Vocabulary	4	215	160	9.98 (0.48)	9.06 (0.61)	0.275*	(0.031, 0.519)	.027	10.9	-0.736 (.462)	4
VCI: Comprehension	5	247	177	9.81 (0.82)	8.81 (0.74)	0.321	(-0.063, 0.704)	.101	66.9	-0.043 (.966)	–
PRI: Block Design	6	260	190	10.84 (0.33)	8.82 (0.75)	0.636**	(0.177, 1.095)	.007	77.4	0.488 (.626)	65
PRI: Picture Completion	4	215	160	9.30 (0.55)	8.26 (0.44)	0.433***	(0.203, 0.663)	<.001	0.0	-1.923 (.055)	13
WMI: Digit Span	6	260	190	9.74 (0.35)	8.96 (0.41)	0.213	(-0.051, 0.476)	.113	35.4	0.994 (.320)	–
WMI: Arithmetic	5	247	177	10.10 (0.56)	8.98 (0.63)	0.334*	(0.056, 0.612)	.019	38.4	-0.236 (.814)	12
PSI: Digit Symbol Coding	6	260	190	7.93 (0.71)	6.73 (0.45)	0.385	(-0.056, 0.826)	.087	76.1	0.851 (.395)	–
Object Assembly	4	215	160	10.25 (0.32)	8.64 (0.80)	0.475*	(0.083, 0.868)	.018	63.8	-0.593 (.553)	20
Picture Arrangement	4	215	160	9.45 (0.84)	7.72 (0.68)	0.672	(-0.054, 1.397)	.070	88.9	-1.318 (.187)	–

ASD, autism spectrum disorder; SZ, schizophrenia; VCI, Verbal Comprehension Index; PRI, Perceptual Reasoning Index; WMI, Working Memory Index; PSI, Processing Speed Index.

For scaled scores, means weighted by group inverse variance are presented with standard errors in parentheses. Scaled scores are age-adjusted to have a sample distribution centered at a mean of 10 and a standard deviation of 3 (range=1-19), with higher scores indicating better performance.

C.I., confidence interval. *p<.05; **p<.01; ***p<.001.

I^2^ (31): percentage of variation across studies that is due to heterogeneity rather than chance.

ERT [Egger’s Regression Test; (32)]: correlation between effect sizes and sample sizes.

Fail-safe n: number of studies with null results that would have to be added to the current set of studies to raise the significance level of the effect size to p=.05 (33).

**Figure 3:**
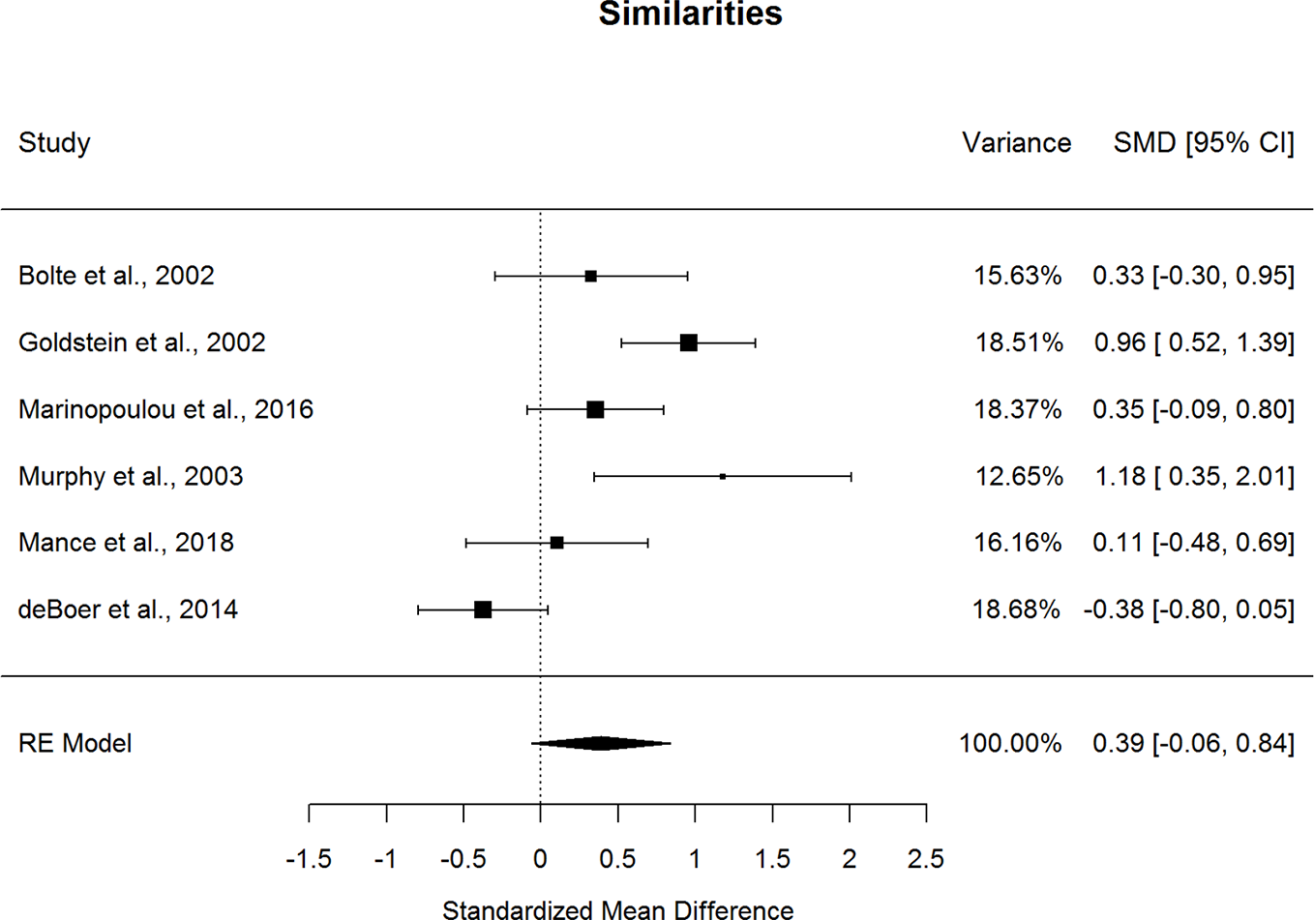
Random effect (re) meta-analysis of standardized mean difference (smd) between autism and schizophrenia groups in similarities scores.

**Figure 4:**
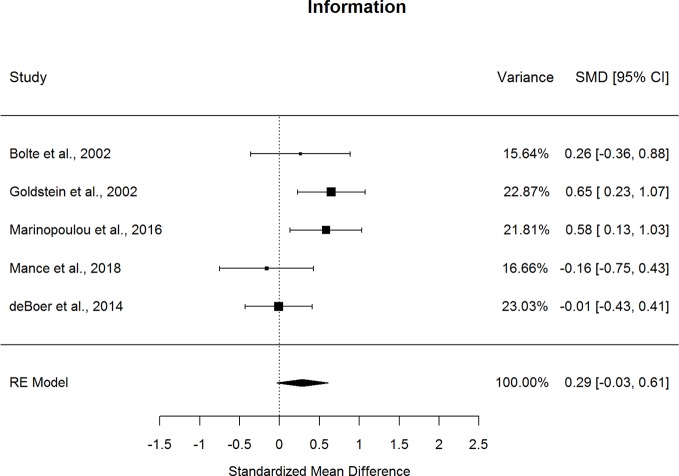
Random effect (re) meta-analysis of standardized mean difference (smd) between autism and schizophrenia groups in information scores.

**Figure 5:**
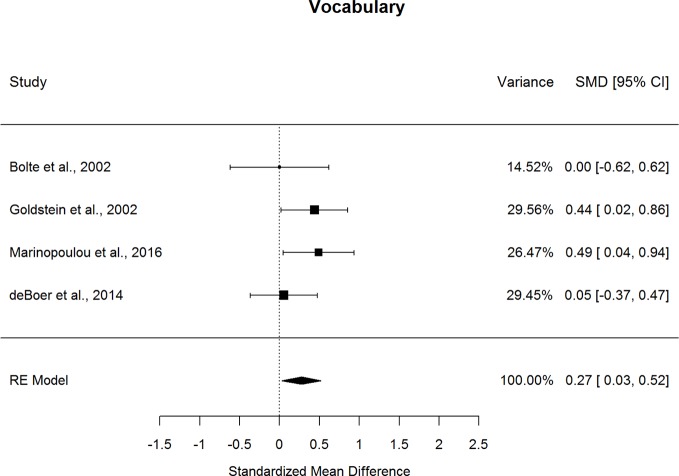
Random effect (re) meta-analysis of standardized mean difference (smd) between autism and schizophrenia groups in vocabulary scores.

**Figure 6:**
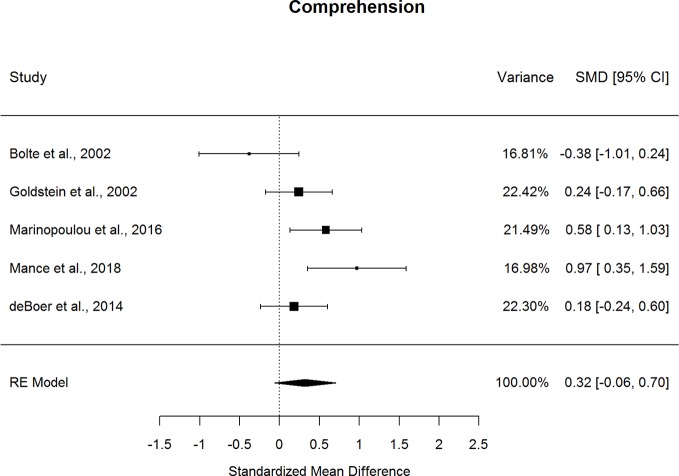
Random effect (re) meta-analysis of standardized mean difference (smd) between autism and schizophrenia groups in comprehension scores.

Forestplots depicting WAIS subtest effect sizes are presented for Similarities (**Figure 3**), Information (**Figure 4**), Vocabulary (**Figure 5**), and Comprehension (**Figure 6**). On average, ASD and schizophrenia participants generally performed within the average range for measures of language and verbal comprehension, with both groups demonstrating the highest scores for Information (scaled score mean=11.84 for ASD and 10.81 for schizophrenia) and the lowest scores for Comprehension (scaled score mean=9.81 for ASD and 8.81 for schizophrenia). Across the four included subtests of Verbal Comprehension, only Vocabulary differed significantly between ASD and schizophrenia, with ASD demonstrating better performance than schizophrenia. The effect size was small (*g*=0.275, *p=*.027) with relatively little heterogeneity across studies that was unlikely to be attributable to chance (*I^2^*=10.9%), suggesting that diagnostic differences in Vocabulary performance are fairly homogenous across studies. Doubling the sample size to include an additional four null-effect studies would be sufficient to render the overall effect nonsignificant (fail-safe number=4), leading us to interpret this result cautiously. The effect size for Vocabulary remained significant after eliminating the only contributing study that included adolescents (13).

Performance on the three other subtests comprising the Verbal Comprehension Index, Similarities, Information, and Comprehension did not differ significantly between ASD and schizophrenia. These nonsignificant findings may be in part due to the substantial heterogeneity across studies observed for these subtest comparisons (*I^2^*=53.5-77.0%). Across all subtests, the ERT was nonsignificant, suggesting that the results are unlikely to be attributable to small-sample bias. Meta-regressions indicated that all results were largely independent of age, sex, intellectual disability, and scale version.

Overall, ASD and schizophrenia show similar performance for assessments of verbal reasoning, general knowledge, and the ability to describe abstract social norms and expressions from early through middle adulthood, with small advantages in ASD compared to schizophrenia for verbal comprehension and expression.

### Diagnostic Comparisons of Visuospatial Abilities and Reasoning and Problem-Solving

**Figure 7:**
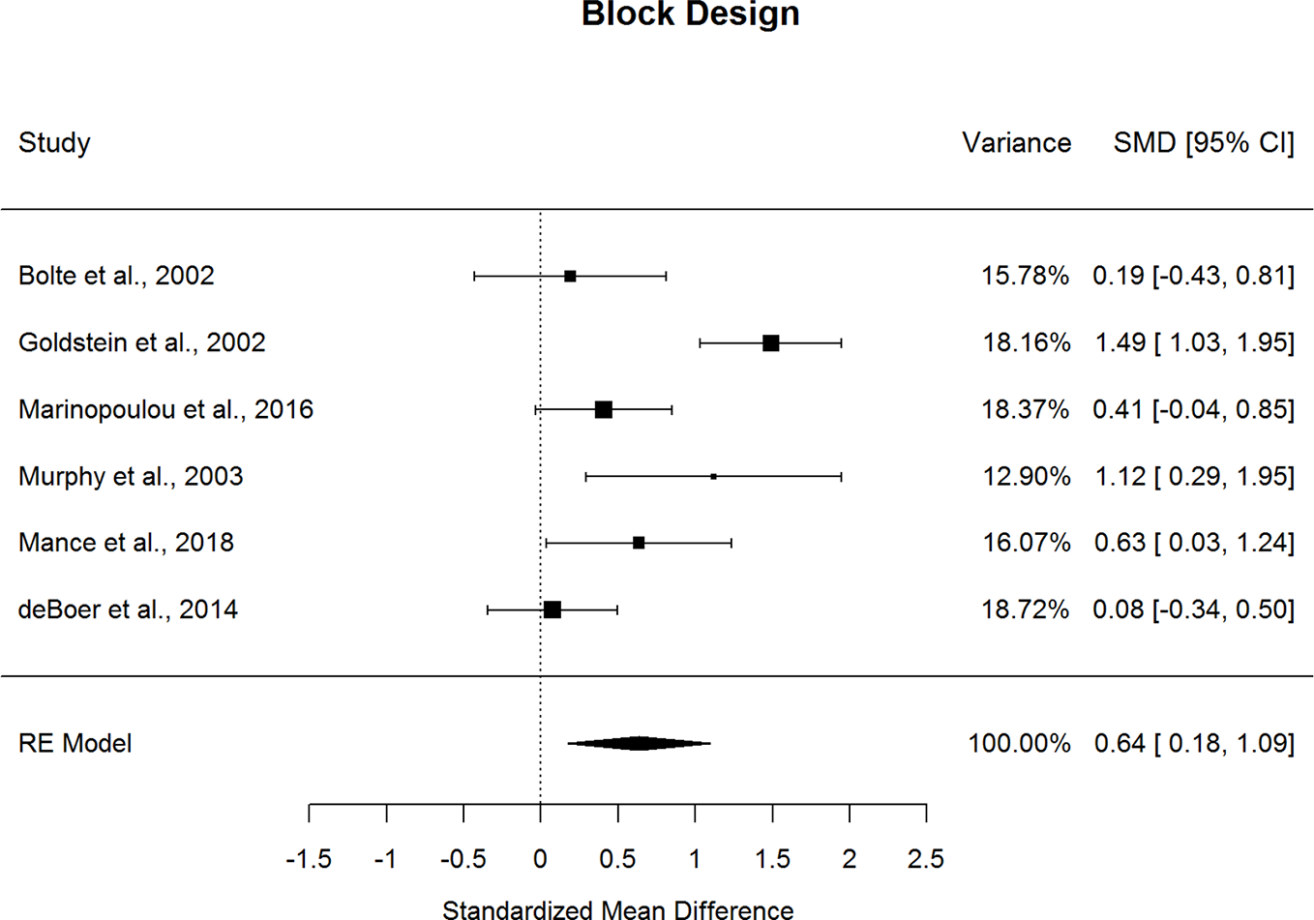
Random effect (re) meta-analysis of standardized mean difference (smd) between autism and schizophrenia groups in block design scores.

**Figure 8:**
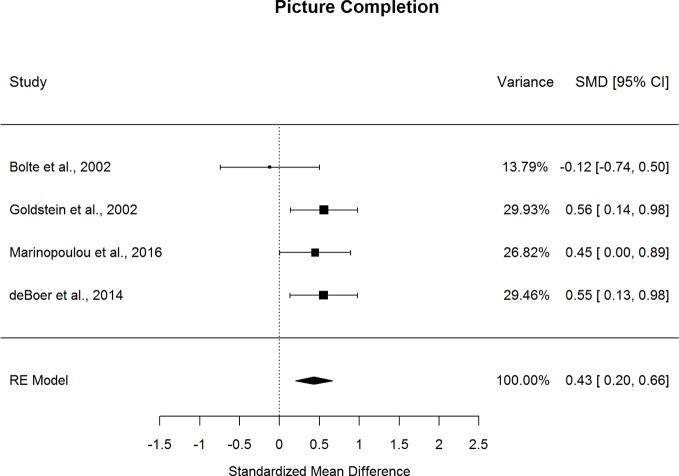
Random effect (re) meta-analysis of standardized mean difference (smd) between autism and schizophrenia groups in picture completion scores.

Forestplots depicting WAIS subtest effect sizes are presented for Block Design (**Figure 7**) and Picture Completion (**Figure 8**). ASD and schizophrenia participants also performed within the average range for measures of visuospatial abilities and reasoning and problem solving, with both groups demonstrating higher scores for Block Design (scaled score mean=10.84 for ASD and 8.82 for schizophrenia) and lower scores for Picture Completion (scaled score mean=9.30 for ASD and 8.26 for schizophrenia). ASD performed better than schizophrenia on both Perceptual Reasoning subtests. Although there was substantial heterogeneity across studies for Block Design (*I^2^*=77.4%), a medium effect size was still observed (*g*=0.636, *p=*.007), with ASD demonstrating substantially better performance than schizophrenia. On the other hand, very little heterogeneity was observed across studies for Picture Completion (*I^2^*=0.0%), for which a medium effect size was observed (*g*=0.433, *p*<0.001), suggesting that ASD consistently showed better performance than schizophrenia for this subtest. Here again, the ERT was nonsignificant, suggesting that small samples were unlikely to bias these results. Furthermore, the fail-safe numbers were substantially larger than the number of included studies, requiring 65 null-effect studies to render the effect size for Block Design nonsignificant, and 13 null-effect studies to render the effect size for Picture Completion nonsignificant. The effect sizes for both Block Design and Picture Completion also remained significant after eliminating the only contributing study that included adolescents (13). Thus, these results are unlikely to change substantially by including many unpublished studies of null effect. These findings were stable across age, sex, intellectual disability, scale version, and matching for age or sex, as indicated by meta-regression results. Overall, these findings indicate that ASD demonstrates strengths in visuospatial processing and reasoning and problem solving compared to schizophrenia from early through middle adulthood.

### Diagnostic Comparisons of Attention and Working Memory

**Figure 9:**
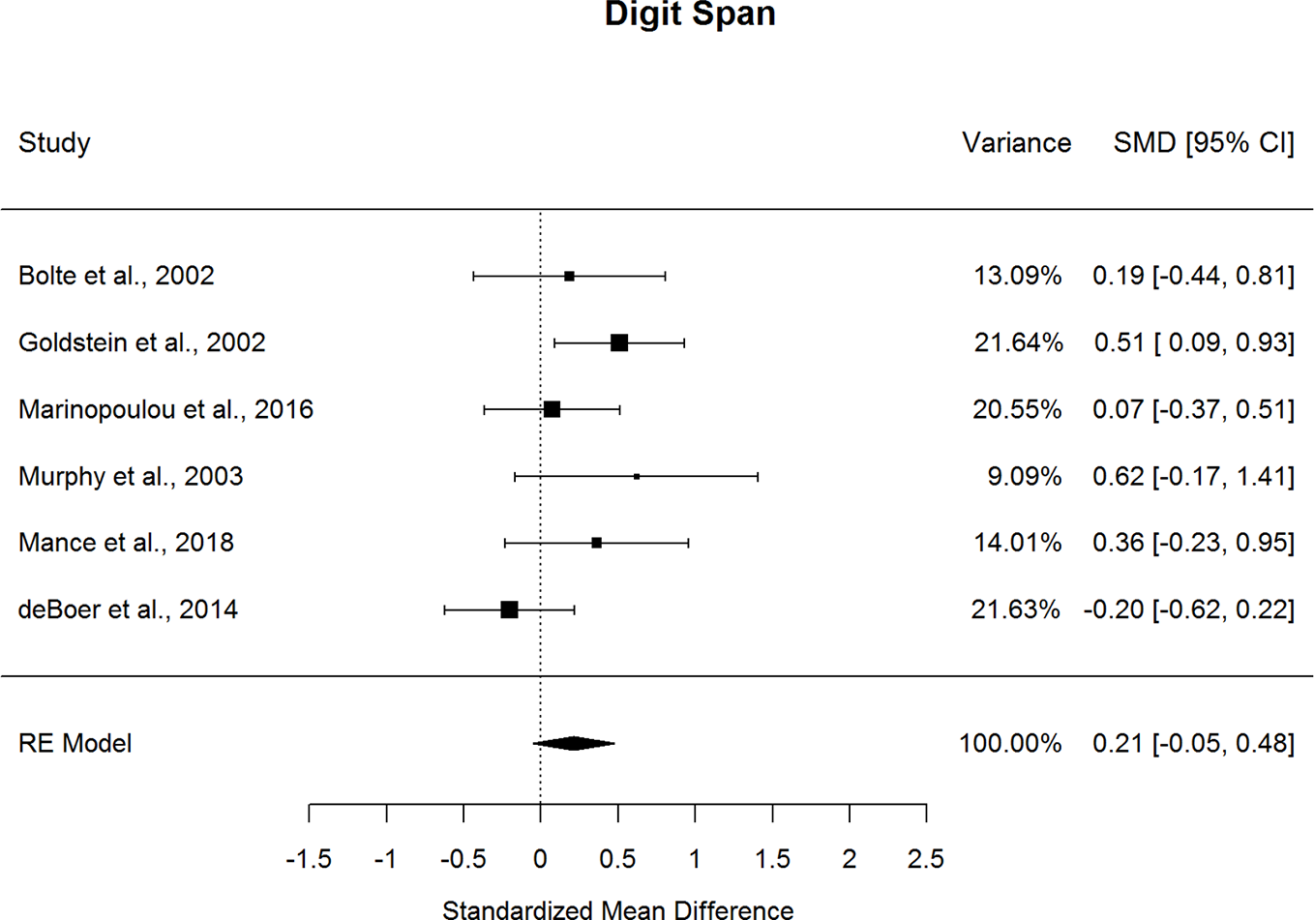
Random effect (re) meta-analysis of standardized mean difference (smd) between autism and schizophrenia groups in digit span scores.

**Figure 10:**
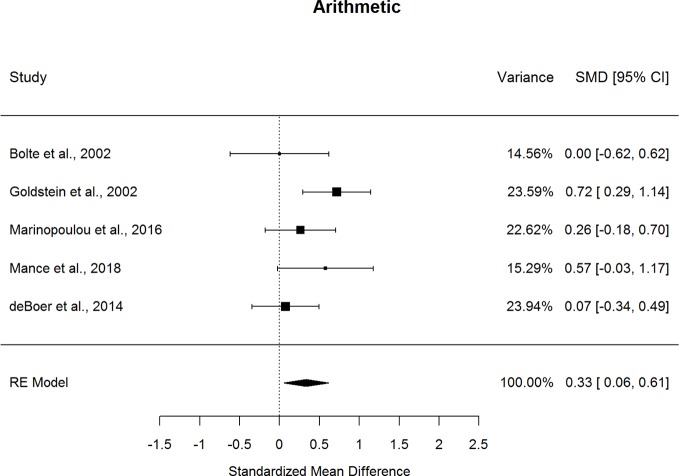
Random effect (re) meta-analysis of standardized mean difference (smd) between autism and schizophrenia groups in arithmetic scores.

Forestplots depicting WAIS subtest effect sizes are presented for Digit Span (**Figure 9**) and Arithmetic (**Figure 10**). On average, ASD and schizophrenia participants generally performed within the average range for measures of attention and working memory, with both groups demonstrating higher scores for Arithmetic (scaled score mean=10.10 for ASD and 8.98 for schizophrenia) and lower scores for Digit Span (scaled score mean=9.74 for ASD and 8.96 for schizophrenia). The findings were mixed for the two Working Memory subtests, despite similar levels of heterogeneity across studies for both subtests (*I^2^*=35.4-38.4%). In particular, ASD and schizophrenia demonstrated similar performance on Digit Span. However, ASD demonstrated better performance than schizophrenia for Arithmetic, with a small effect size (*g*=0.334, *p=*.019). The ERT was nonsignificant for both subtests, suggesting a lack of small-sample bias. With a reasonably large fail-safe number of 12 compared to the number of contributing studies (*k*=5), the finding for Arithmetic is unlikely to change even by doubling the sample size by including additional null-effect studies. Similarly, even after eliminating the only contributing study that included adolescents (13), ASD demonstrated better Arithmetic performance than schizophrenia. The results were not moderated by age, sex, IQ, intellectual disability, scale version, or matching for age or sex. Overall, these findings suggest that ASD and schizophrenia show some differences in working memory performance from early to middle adulthood.

### Diagnostic Comparisons of Processing Speed

**Figure 11:**
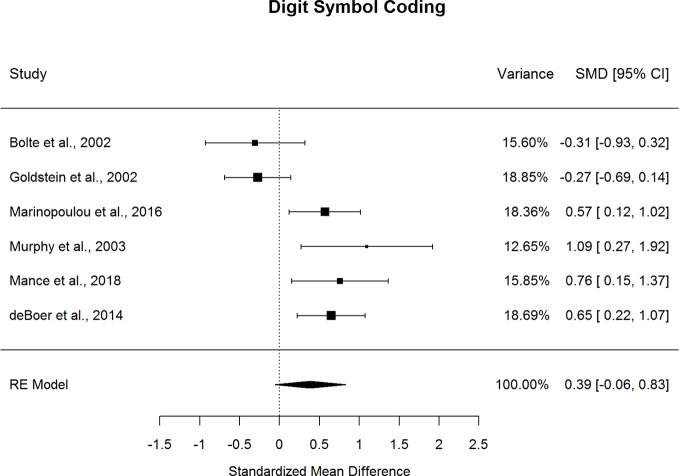
Random effect (re) meta-analysis of standardized mean difference (smd) between autism and schizophrenia groups in digit symbol coding scores.

Forestplots depicting WAIS subtest effect sizes are presented for Digit Symbol Coding (**Figure 11**). ASD and schizophrenia participants performed within the borderline range for the included measure of processing speed (scaled score mean=7.93 for ASD and 6.73 for schizophrenia). ASD and schizophrenia did not demonstrate significant differences in Digit Symbol Coding performance. However, the magnitude of the differences varied by mean sample age but not by other moderators, such that ASD demonstrated greater advantages in Digit Symbol Coding performance over schizophrenia in middle adulthood compared to early adulthood (*t*(6)=-2.595, *p=*.009, *I^2^*=24%, Adjusted *R^2^*=90%). This suggests that differences between ASD and schizophrenia in processing speed may change with age, with strengths for ASD relative to schizophrenia increasing with age.

### Diagnostic Comparisons of Abilities Associated with Social Cognition

**Figure 12:**
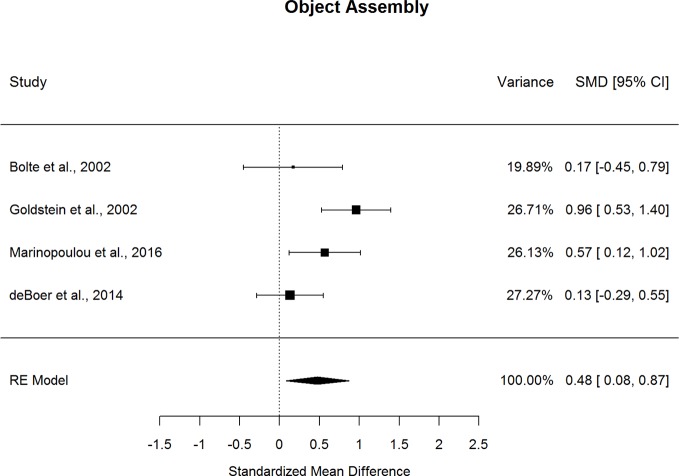
Random effect (re) meta-analysis of standardized mean difference (smd) between autism and schizophrenia groups in object assembly scores.

**Figure 13:**
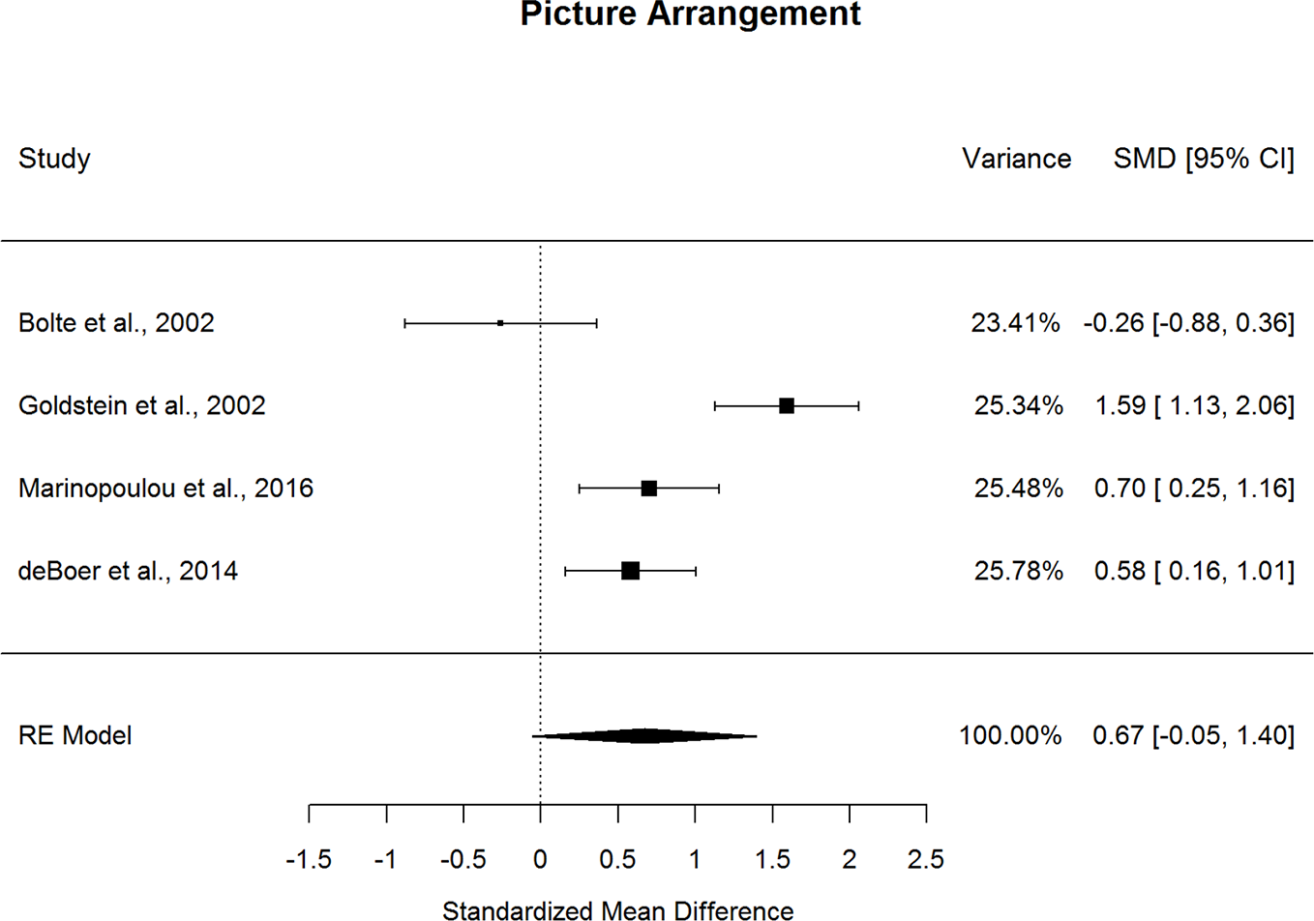
Random effect (re) meta-analysis of standardized mean difference (smd) between autism and schizophrenia groups in picture arrangement scores.

Forestplots depicting WAIS subtest effect sizes are presented for Object Assembly (**Figure 12**) and Picture Arrangement (**Figure 13**). On average, ASD and schizophrenia participants performed within the normal range for abilities associated with social cognition, with higher scores for Object Assembly (scaled score mean=10.25 for ASD and 8.64 for schizophrenia) than Picture Arrangement (scaled score mean=9.45 for ASD and 7.72 for schizophrenia). Of the two additional subtests, which have been found to tap into social cognition (26), ASD performed better than schizophrenia on Object Assembly (*g*=0.475, *p=*.018) but demonstrated similar performance to schizophrenia for Picture Arrangement. The fail-safe number of 20 for Object Assembly suggests that this finding is unlikely to change unless at least 20 additional null-effect studies were included. Furthermore, ASD showed better performance than schizophrenia for Object Assembly even after removing the only contributing study that included adolescents (13). Both subtests showed considerable heterogeneity across studies (*I^2^*=63.8-88.9%) and were unlikely to be biased by small-sample studies. Furthermore, the results were not moderated by age, sex, IQ, intellectual disability, scale version, or matching for age or sex.

## Discussion

To our knowledge, this is the first study to systematically review comparisons of nonsocial cognition between ASD and schizophrenia, consolidating all reports to date of WAIS subtest comparisons across 450 participants. Across domains, ASD and schizophrenia demonstrated generally comparable performance on processing speed and verbal comprehension. In contrast, ASD demonstrated better performance than schizophrenia for visuospatial processing and reasoning and problem-solving (*g*=0.636), followed by visual attention and organization (*g*=0.433-0.475), working memory (*g*=0.334) and language (*g*=0.275). Overall, although ASD and schizophrenia perform similarly across many subtests, where these neurodevelopmental disorders diverge in nonsocial cognitive functioning, ASD consistently bears advantages over schizophrenia. Even for the subtests which did not show statistically significant differences between ASD and schizophrenia, all effect sizes were in the same direction, with ASD tending to demonstrate better performance than schizophrenia.

This study substantially extends prior literature investigating nonsocial cognition within each neurodevelopmental disorder. In particular, our findings are consistent with effect size estimates across separate meta-analyses examining ASD or schizophrenia relative to neurotypical controls, which suggested working memory (Arithmetic), visuospatial processing, reasoning and problem solving (Block Design, Picture Completion, and Object Assembly), and language (Vocabulary) as cognitive domains in which ASD may demonstrate better performance than schizophrenia (8, 9). Of the five cognitive measures for which ASD showed better performance compared to schizophrenia, the most prominent differences were observed for three measures of visuospatial abilities: Block Design, Picture Completion, and Object Assembly. This suggests that, though the autism participants in all the contributing studies are verbal, they demonstrate comparable difficulties to schizophrenia in verbal abilities and show less severe difficulties with visuospatial abilities. To the extent that these social cognitive abilities rely upon visual perception and organization of nonsocial stimuli (26), ASD may demonstrate some advantages over schizophrenia in social cognition.

This pattern of cognitive performance differences was largely consistent across key demographic variables. The only meta-regression that reached statistical significance was the relationship between mean sample age and Digit Symbol Coding, a processing speed measure. Here, ASD showed better performance than schizophrenia with increasing age from late adolescence to middle adulthood, suggesting that the divergence in Digit Symbol Coding performance between ASD and schizophrenia becomes more pronounced with age. Notably, Digit Symbol Coding is the measure that most highly differentiates schizophrenia from controls of all neuropsychological measures (40). Autism demonstrates minimal age-related changes in WAIS measures of processing speed, including Digit Symbol Coding, from ages 6 through 39 (41). In contrast, schizophrenia demonstrates substantial declines in Digit Symbol Coding between late childhood (age 7-13) and middle adulthood (age 38) (42). Bearing in mind that the age ranges for these studies extend earlier into childhood, age-related changes in processing speed differences between ASD and schizophrenia may be attributable to stable performance across age in autism and declining performance with age in schizophrenia.

Other than age-related changes in the difference between ASD and schizophrenia for Digit Symbol Coding, diagnostic comparisons for nonsocial cognition were not attributable to age, sex, intellectual disability, scale version, IQ, nor group differences in age and sex. Given the wide age range of the groups, with a mean age ranging from 16 to 41, and the diverse sex ratios, ranging from equal sex ratios to completely male, this suggests that similarities and differences in nonsocial cognitive performance are largely consistent from early through middle adulthood across sexes for these neurodevelopmental disorders. Notably, intellectual disability was not a significant moderator of the findings, supporting the generalizability of these diagnostic patterns of relative strengths and weaknesses in nonsocial cognition to individuals with substantial impairments in general cognitive ability. Furthermore, given that IQ did not differ significantly between groups within most studies and that IQ was not a significant moderator for any of the findings, the mean group differences in subtest performance likely reflect relative strengths and weaknesses in specific cognitive domains rather than differences in general cognitive ability.

Taken together, the magnitude of similarities and differences between ASD and schizophrenia in nonsocial cognition differs across domains, with differences being most obvious for visuospatial abilities and reasoning and problem-solving, and to a lesser extent, working memory and language. The current review extends prior literature comparing other cognitive domains between ASD and schizophrenia, notably, social cognition (12). In the current meta-analysis, we identified multiple nonsocial cognitive domains in which ASD and schizophrenia demonstrate significantly different performance levels. In contrast, ASD does not show significant differences from schizophrenia in social cognitive domains, as described in the most recent meta-analysis (12). It should be noted that three to eight studies contributed to each social cognitive domain analyzed in the prior meta-analysis (12), comparable to the sample sizes for the nonsocial cognitive measures analyzed in the current meta-analysis. However, a wide range of measures were consolidated for each social cognitive domain in the meta-analysis of social cognitive measures (12), whereas measures were analyzed separately across similar versions of a standardized cognitive battery in this meta-analysis of nonsocial cognitive measures, thereby reducing method heterogeneity and likely increasing our ability to detect group differences in nonsocial cognition. Indeed, here we found that ASD demonstrated better performance than schizophrenia for two visuospatial processing measures that comprise a social cognition factor (26). This further suggests that group differences in social cognitive performance may vary depending on whether the social cognitive measures used rely heavily on visuospatial processing abilities, which may have been a source of methodological heterogeneity in the prior meta-analysis of social cognition (12). Our findings therefore suggest the importance of examining not only social cognition, but also nonsocial cognition, to gain a fuller picture of cognitive functioning performance across ASD and schizophrenia.

### Considerations

Overall, our findings emphasize the importance of examining nonsocial cognitive processes in addition to social cognitive processes across ASD and schizophrenia. Building upon this work, we may investigate whether these transdiagnostic similarities and differences in cognitive functioning may arise from shared biological processes and may be remediated by similar strategies (43). Despite the pathophysiological and treatment implications of our findings, certain limitations should be considered. In particular, measurement equivalence is constrained across WAIS versions, most notably due to the Flynn effect, whereby group mean IQ scores increase over time (44). Relevant to the scale versions that are most frequently used in the current study, the largest discrepancies in how a given group may perform across the WAIS-R (i.e. the second edition of the WAIS) and the WAIS-III are observed for timed subtests, including Object Assembly and Coding, whereas the smallest differences are observed for untimed subtests, including Digit Span and Information (45). The authors of the contributing studies generally did not report when data were collected in relation to the availability of the most recent scale version. However, given that the effect sizes that we meta-analyzed were based on the difference between ASD and schizophrenia groups *within* a WAIS subtest version rather than *across* WAIS subtest versions, to the extent that both groups are similarly impacted by measurement changes across scale versions, our results are unlikely to be systematically biased by measurement error to the Flynn effect and/or to other sources of measurement variance across WAIS versions (44, 46). This interpretation is further supported by our finding that scale version did not moderate mean group differences in cognitive performance. In addition, although each contributing study contributed multiple cognitive measures, the meta-analyses would benefit from more studies contributing to each measure. Because the WAIS does not include measures of visual or verbal memory, we were unable to examine cognitive functioning in these domains. Thus, this work may be further expanded upon by using transdiagnostically validated cognitive batteries that include measures of memory, such as the MATRICS Consensus Cognitive Battery (47).

We acknowledge additional considerations beyond measurement in interpreting our findings. Specifically, some studies included participants with high-functioning autism whereas other samples included participants with a range of autism severity. Likely due to the inclusion of verbal adults with ASD, the group mean IQ across the ASD groups in the meta-analyses is comparable to that of normative samples (i.e. with a mean score approximating 100 and standard deviation approximating 10). Our study inclusion criteria for the ASD group reflects the strong selection bias in autism research, where approximately 94% of participants with ASD do not have an intellectual disability for studies published in autism-specific journals in 2016, and only 2% of ASD participants in these studies are minimally or non-verbal (48). Along with this heterogeneity in severity, although ASD often co-occurs with other disorders, such as attention-deficit/hyperactivity disorder, the included studies did not account for these diagnoses. Finally, given the substantial differences across the included studies in the clinical characterization of the groups, we were unable to examine clinical and functional moderators of group differences in nonsocial cognition. Our study therefore provides a rationale for recommending the use of consistently validated measures of cognitive ability, the inclusion of participants across the full range of verbal and intellectual abilities in ASD and schizophrenia, and the careful characterization of clinical features across ASD and schizophrenia.

### Implications

In summary, ASD and schizophrenia demonstrate some overlapping and distinctive patterns of cognitive functioning, with similar performance on processing speed, attention, and verbal comprehension and ASD performing better than schizophrenia on working memory, language, and especially, visuospatial perception and reasoning. These findings are consistent with and substantially extend prior meta-analyses of case-control studies for ASD and schizophrenia (8, 9). Our findings thus highlight the importance of going beyond investigating social cognition in ASD and schizophrenia to characterize nonsocial cognition across these neurodevelopmental disorders. Ultimately, this review provides a launching point from which we can develop and adapt transdiagnostic strategies to bolster cognitive functioning across ASD and schizophrenia.

## Data Availability Statement

The raw data supporting the conclusions of this article will be made available by the authors, without undue reservation, to any qualified researcher.

## Author Contributions

SK contributed conception and design of the study, conducted the literature review, extracted the data, organized the database, performed the statistical analysis, and wrote the first draft of the manuscript. SE provided substantive feedback on the analysis and interpretation of the results. All authors contributed to manuscript revision, read and approved the submitted version.

## Funding

This work was supported by the Canadian Institutes of Health Research (SK, Doctoral Foreign Study Award) and National Institutes of Health (SE, MH-92440, MH-8585, MH-95783, RR- 24154, and DA-30763); Autism Speaks (SE, 5703); and the Department of Defense (SE, AR100344).

## Conflict of Interest

The authors declare that the research was conducted in the absence of any commercial or financial relationships that could be construed as a potential conflict of interest.
